# Characterization of the first mitogenomes of the smallest fish in the world, *Paedocypris progenetica*, from peat swamp of Peninsular Malaysia, Selangor, and Perak

**DOI:** 10.5808/gi.21081

**Published:** 2022-03-31

**Authors:** NorJasmin Hussin, Izzati Adilah Azmir, Yuzine Esa, Amirrudin Ahmad, Faezah Mohd Salleh, Puteri Nur Syahzanani Jahari, Kaviarasu Munian, Han Ming Gan

**Affiliations:** 1 Faculty of Applied Sciences, Universiti Teknologi MARA (UiTM), 40450 Shah Alam, Malaysia; 2 School of Biology, Faculty of Applied Sciences, Universiti Teknologi MARA (UiTM) Negeri Sembilan, Kampus Kuala Pilah, Pekan Parit Tinggi, 72000 Kuala Pilah, Malaysia; 3 Department of Aquaculture, Faculty of Agriculture, Universiti Putra Malaysia, 43400 Serdang, Malaysia; 4 School of Marine and Environmental Sciences, Universiti Malaysia Terengganu, 21030 Kuala Nerus, Malaysia; 5 Institute of Tropical Biodiversity and Sustainable Development, Universiti Malaysia Terengganu, 21030 Kuala Nerus, Malaysia; 6 Department of Biosciences, Faculty of Science, Universiti Teknologi Malaysia, Johor, Johor Bahru 81310, Malaysia; 7 Forest Biodiversity Division, Forest Research Institute Malaysia, Kepong 52109, Malaysia; 8 GeneSEQ Sdn Bhd, Bukit Beruntung, 48300 Rawang, Malaysia

**Keywords:** mitogenome, *Paedocypris progenetica*, Peninsular Malaysia

## Abstract

The two complete mitochondrial genomes (mitogenomes) of *Paedocypris progenetica*, the smallest fish in the world which belonged to the Cyprinidae family, were sequenced and assembled. The circular DNA molecules of mitogenomes P1-*P. progenetica* and S3-*P. progenetica* were 16,827 and 16,616 bp in length, respectively, and encoded 13 protein-coding genes, 22 transfer RNA genes, two ribosomal RNA genes, and one control region. The gene arrangements of *P. progenetica* were identical to those of other *Paedocypris* species. BLAST and phylogenetic analyses revealed variations in the mitogenome sequences of two *Paedocypris* species from Perak and Selangor. The circular DNA molecule of *P. progenetica* yield a standard vertebrate gene arrangement and an overall nucleotide composition of A 33.0%, T 27.2%, C 23.5%, and G 15.5%. The overall AT content of this species was consistent with that of other species in other genera. The negative GC-skew and positive AT-skew of the control region in *P. progenetica* indicated rich genetic variability and AT nucleotide bias, respectively. The results of this study provide genomic variation information and enhance the understanding of the mitogenome of *P. progenetica*. They could later deliver highly valuable new insight into data for phylogenetic analysis and population genetics.

## Introduction

The *Paedocypris* populations are rapidly declining worldwide due to anthropogenic and environmental actions that pose a threat to their survival [1]. According to Sam et al. [2], the evolution of small sizes, or miniaturization, is extensively seen in vertebrate species and is most commonly documented in amphibians and fishes. Southeast Asia harbors highly acidic blackwater peat swamps that serve as habitats for miniature fish, which are nearly all endemic to these habitats. The features of miniature phenotypes exhibit morphological novelty and increased morphological variability and are mostly unique combinations of ancestral phenotypes that are derived through structural simplification and reduction [3]. Interestingly, the smallest fish in the world, *Paedocypris progenetica*, is found in Peninsular Malaysia. However, the lack of its genomic data in GenBank could hinder the extensive study of this remarkable species. The mitochondrial genome (mitogenome) contains multiple genes that are noteworthy for ecological and evolutionary studies to investigate the phylogeny and biodiversity of complex species by using high-throughput sequencing technologies [4]. Hence, this study provided the whole mitogenome of *P. progenetica* from Peninsular Malaysia for the first time.

## Methods

### DNA sampling and sequencing

The samples of *P. progenetica* were collected from North Peat Swamp Selangor (3.39ʹN, 101.15ʹʹE) and Pondok Tanjung Perak (5.04ʹN, 100.4ʹʹE), Peninsular Malaysia in February 2021. Genomic DNA was extracted from the tissue of *P. progenetica* specimens by using a ReliaPrep gDNA Tissue Miniprep system (Promega, Madison, WI, USA), fragmented with a Bioruptor system, and the remaining tissue is currently deposited at University Putra Malaysia (UPM). The library was prepared by using a NEBNext Ultra II DNA Library Prep Kit for Illumina in accordance with the manufacturer’s protocol. The sample was then sequenced by using an Illumina NovaSeq 6000 platform (Illumina, San Diego, CA, USA) with 150 paired-end modes (PE150) [5].

### Mitogenome assembly, annotation, and sequence analysis

Sequencing adapters, low-quality stretches, and leading/tailing Ns were trimmed from the raw reads of the sequences by using AdapterRemoval V2.2.2 [6]. Forward and reverse reads were interleaved into single file and the assembly were carried out using two different softwares, NOVOPlasty v4.2 [5] and Megahit v1.2.9 [7], both using default k-mer sizes. For the assembly using NOVOPlasty, the reference mitogenome of the closest species in GenBank was used as the seed reference. Subsequently, quality was evaluated by utilizing the PALEOMIX pipeline. Gene identification and tRNA structure prediction were performed with the Mitochondrial Genome Database of Fish (http://mitofish.aori.u-tokyo.ac.jp/). The complete mitogenomes were then annotated, and a circular mitogenome map was generated by using MitoAnnotator [8]. Additionally, the nucleotide composition of the mitogenome was determined by applying MEGA v. 7.0 [9]. Nucleotide compositional differences were determined by using the formulae AT skew = (A − T)/(A + T) and GC skew = (G − C)/ (G + C) [10], where each nucleic base letter represents the count of a specific base.

### Phylogenetic analysis

A phylogenetic tree of *Paedocypris* mitogenomes, including the sequences retrieved from GenBank, was constructed by using MEGA v. 7.0 software [10], which contains advanced methods and tools for phylogenomics and an optimized 64-bit computing system for the analysis of a large dataset. The neighbor-joining method [11] was utilized for the comparative analyses of the nucleotide sequences of *P. progenetica*, comprising 13 protein-coding genes (PCGs), and those of putative *Paedocypris* species, including six sequences from the GenBank database. The 13 PCGs (without stop codons) were aligned with the vertebrate mitochondrial genetic code by using the MASCE [12] algorithm in PhyloSuite 1.2.2 [13]. The alignments of each individual gene were concatenated as different datasets with six mitogenomes retrieved from the GenBank entry. The multiple alignments of the concatenated nucleotide sequences of the 13 PCGs were conducted by using the MEGA v. 7.0 program [14]. The bootstrap confidence of 1,000 replicates was applied to evaluate the resulting phylogenetic tree. The trees were rooted by using the GenBank entry of the closely related family of *Schismatorhynchos nukta* (Cyprinidae) as an outgroup.

## Results and Discussion

The complete mitogenome sequences of S3-*P. progenetica* (OK413207) and P1-*P. progenetica* (OK356905) were 16,874 and 16,616 bp, respectively, as shown in Table 1. The two recorded mitogenomes were parallel to the *Serranochromis robustus* and *Buccochromis nototaenia* fish mitogenomes and comprised of 13 PCGs, 22 transfer RNA genes, two ribosomal RNA genes, and one control region (Table 2) that were clearly within the range [15]. According to Sun et al. [14] and Mullens et al. [16], a set of 13 PCGs and two rRNAs in the mitochondrial gene are consistently used as markers to strengthen the identification or resolve high-level relationships between fish species. The stipulated data in Table 2 indicated that the ND6 and seven tRNA genes (tRNA^ile^, tRNA^Ala^, tRNA^Asn^, tRNA^Cys^, tRNA^Ser^, tRNA^Glu^, and tRNA^Pro^) were encoded on the L-strand, whereby, most of *P. progenetica* mitochondrial genes were encoded on the H-strand. This finding was consistent with the result reported by Sam et al. [2] on the mitogenomes of *P. micromegethes* and *P. carbunculus* as there were no significant changes found between the populations of *P. progenetica* between the conserved genes (PCGs, tRNAs and rRNAs). However, the mitogenome length of *P. micromegethes* and *P. carbunculus* were clearly different from those of *P. progenetica*, presumably because of the variations in the control region (D-loop).

### Phylogenetic relationship

**Fig. 1.**
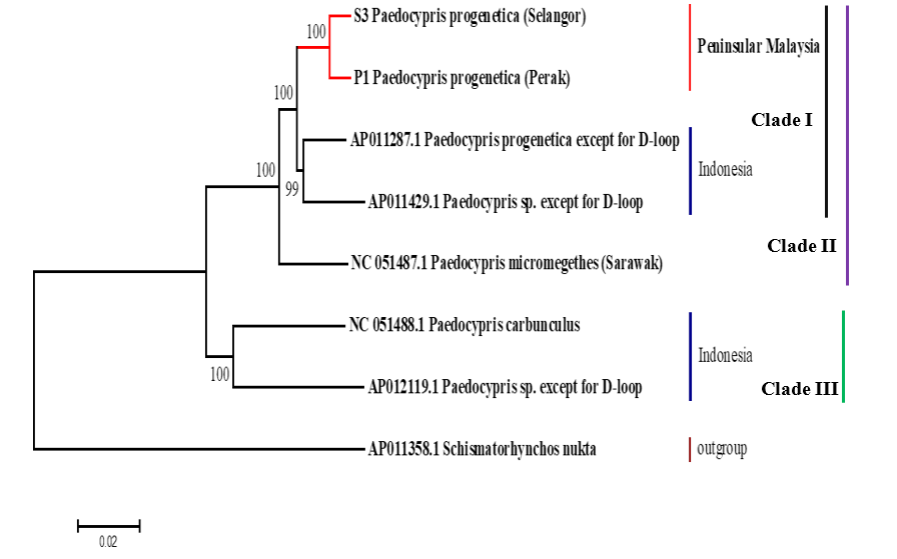
The phylogenetic tree of *Paedocypris progenetica* mitogenomes (OK356905 and OK413207) and other *Paedocypris* species available in GenBank. The bootstrap values were indicated in each branch of the tree representing the result of neighbor-joining probability. *Shcimatorhynchos nukta* was selected as an outgroup.

The mitogenomic phylogeny analysis clustered the two mitogenomes of *P. progenetica* (OK356905 and OK413207) with the mitogenome of *P. progenetica* from Indonesia (AP011287) [17] and rooted them with the mitogenomes of other *Paedocypris* species [2,18] (Fig. 1) with the high support of 100% bootstrap and 1.00 posterior probability. GenBank revealed that the closest match (>96% similarity) was between the newly sequenced mitogenomes of *P. progenetica* from Peninsular Malaysia and the mitogenomes of *P. progenetica* (AP011287) from a peat swamp in Sumatera, Indonesia [18] (Table 1). Moreover, <96% similarity was found among *Paedocypris* species. The molecular evidence strongly indicated that Clade 1, which included the *P. micromegethes* (NC_051487.1) subclade-1, comprised a stable monophyletic group. The latest research has found identical ancestral patterns for *Cirrhirnus reba*, which aligned in the same clade containing the same species [19].

### Protein-coding genes

The prominent features of *Paedocypris* mitochondrial genes are listed in Table 3, which indicates that all PCGs, except for the COI gene that began with GTG, began with the start codon (ATG). The seven PCGs including nad1, cox1, atp8, atp6, nad4l, nad4, and nad5 were terminated by a complete and canonical stop codon (TAA or TAG). However, the genes encoding cox2, cox3, nad2, and nad3 had a truncated stop codon. Similar to the finding reported by Sam et al. [2], except for the COI gene that was terminated by GTG, most PCGs in the mitogenomes of *P. carbunculus* and *P. micromegethes* were terminated by the codon TAR (TAA/TAG) or an incomplete codon (TA-/T--). According to Zhong et al. [20], a truncated stop codon (T) is commonly found in the mitochondrial gene of metazoans, such as the spider *Habronattus oregonensis*, and does not affect mitochondrial gene transcription or translation because the complete stop codon is presumably obtained through post-transcriptional polyadenylation [21].

### Gene arrangements

The remarkable species *P. progenetica* of both samples (S3 and P1) from Peninsular Malaysia were aligned with 96% similarity of *P. progenetica* collected from Indonesia (AP011287) retrieved in GenBank entry. The overall nucleotide composition of *P. progenetica* was 33.0% A, 27.2% T, 23.5% C, and 15.5% G and showed a slightly AT-rich region (60.25%); these results were consistent with the patterns found in most fish mitogenomes [22]. The nucleotide composition of the *P. progenetica* mitogenome was highly biased toward A + T and had similar values as other *Paedocypris* species, such as *P. progenetica* from Banka. The PCGs had a slightly higher A + T content (61.7%) compared to ribosomal RNA genes (59.8%). Based on Table 3, the AT and GC skew of *P. progenetica* showed 0.10 and −0.20, respectively. The GC skews of all genes, except for those of NAD6 and tRNA, which were positive for both populations, were negative and indicated a regular pattern of base composition behavior in the *P. progenetica* mitogenome. This result agreed well with that of Sam et al. [2], who discovered the AT-skew was mainly positive and the GC-skew were mostly negative values in distinct gene regions of the *P. micromegethes* and *P. carbunculus* mitogenomes.

Meanwhile, a vast difference in nucleotide composition in the control region (D-loop region) located between *trnP* (tRNA^Phe^) and *trnF* (tRNA^Pro^) can be seen in this genus. The lengths of PCGs, tRNAs, and rRNAs were conserved, and the variations were mainly attributed to the control region. The lengths of the D-loop region in *P. micromegethes* and *P. carbunculus* were 1,590 and 1,662 bp, respectively, whereas we found the considerably shorter D-loop region length of 1,209 bp (OK356905 and OK413207). These results differed because the D-loop region exhibits a rapid evolutionary rate and tends to possess lower purifying selection compared to PCGs that amass variations in length [23]. According to Li et al. [24], noncoding regions in metazoan mitogenomes frequently vary in length from species to species. However, the D-loop region of the reference species collected from Indonesia (AP011287) was not recorded in GenBank. Eventually, future studies on the noncoding region may contribute genetic data and enhance studies on the genomic data of *P. progenetica*.

## Conclusion

The full mitogenome sequence of *P. progenetica* was analyzed and compared with that of other *Paedocypris* species mainly focused on *P. micromgethes* and *P. carbunculus* in the Cyprinidae family. The mitogenome length of *P. progenetica* was shorter compared to other *Paedocypris* species predominantly due to variations in the D-loop region. The comparison of the complete mitogenome data and phylogenetic relationships of *Paedocypris* species provided fundamental information for evolutionary biology and are particularly important for future studies using the D-loop region and whole-genome sequences to resolve the relationship among *Paedocypris* species fully.

## Figures and Tables

**Table 1.** The reported mitogenome of *Paedocypris progenetica* from Peninsular Malaysia

Sample ID	Genbank accession number	Origin	Length (bp)	Sex	GC%	AT%
P1	OK356905	Pondok Tanjung	16,827	Male	38.3	61.7
		Perak, Malaysia				
S3	OK413207	North Peat Swamp	16,616	Female	38.5	61.5
		Selangor, Malaysia				

**Table 2.** Gene features of the mitochondrial genome of *Paedocypris progenetica*

Gene	Position		Size (bp)	Start/Stop codon	Strand
	From	To			
*tRNA-Phe*	1	69	69		+
*12S rRNA*	70	1018	949		+
*tRNA-Val*	1019	1090	72		+
*16S rRNA*	1091	2762	1,672		+
*tRNA-Leu*	2763	2837	75		+
*ND1*	2838	3812	975	ATG/TAA	+
*tRNA-Ile*	3813	3882	70		+
*tRNA-Gln*	3881	3951	71		‒
*tRNA-Met*	3952	4020	69		+
*ND2*	4021	5059	1,039	ATG/T	+
*tRNA-Trp*	5060	5130	71		+
*tRNA-Ala*	5133	5201	69		‒
*tRNA-Asn*	5203	5275	73		‒
*tRNA-Cys*	5310	5374	65		‒
*tRNA-Tyr*	5375	5444	70		‒
*COI*	5446	6990	1,545	GTG/TAG	+
*tRNA-Ser*	6994	7064	71		‒
*tRNA-Asp*	7066	7138	73		+
*COII*	7152	7842	691	ATG/T	+
*tRNA-Lys*	7843	7915	73		+
*ATPase-8*	7917	8084	168	ATG/TAA	+
*ATPase 6*	8075	8757	683	ATG/TA(A)	+
*COIII*	8758	9541	784	ATG/T	+
*tRNA-Gly*	9542	9613	72		+
*ND3*	9614	9959	346	ATG/TAG	+
*tRNA-Arg*	9960	10030	71		+
*ND4L*	10031	10327	297	ATG/TAA	+
*ND4*	10321	11702	1,382	ATG/TA(A)	+
*tRNA-His*	11703	11771	69		+
*tRNA-Ser*	11772	11840	69		+
*tRNA-Leu*	11842	11914	73		+
*ND5*	11915	13750	1,836	ATG/TAG	+
*ND6*	13747	14268	522	ATG/TAA	‒
*tRNA-Glu*	14270	14338	69		‒
*Cyt b*	14341	15477	1,137	ATG/TAA	+
*tRNA-Thr*	15480	15550	71		+
*tRNA-Pro*	15549	15618	70		‒
*Control Region*	15619	16728	1,209		

**Table 3.** The composition and skewness between *P. progenetica* from *Peninsular Malaysia,* Malaysia and Sumatera, Indonesia

Feature	A + T%		AT skew		GC skew	
	Indonesia	P.M	Indonesia	P.M	Indonesia	P.M
Whole genome	61.22	61.1	0.1	0.1	‒0.20	‒0.20
PCGs	61.8	61.7	0.04	0.03	‒0.28	‒0.22
*nad1*	59.1	59.3	0.05	0.05	‒0.29	‒0.29
*nad2*	65.1	64.5	0.18	0.18	‒0.40	‒0.41
*cox1*	59.9	59.4	‒0.05	‒0.05	‒0.10	‒0.10
*cox2*	62.1	61.2	0.09	0.1	‒0.18	‒0.18
*atp8*	64.3	65.5	0.11	0.1	‒0.37	‒0.64
*atp6*	64.3	63.1	‒0.06	0.06	‒0.31	‒0.30
*cox3*	57.7	57.9	0.03	0.02	‒0.23	‒0.22
*nad3*	61.3	61.2	‒0.02	0.00	‒0.22	‒0.24
*nad4l*	61	59.9	‒0.01	‒0.02	‒0.30	‒0.29
*nad4*	63.1	64.2	0.10	0.11	‒0.22	‒0.23
*nad5*	62.5	62.6	0.11	0.12	‒0.27	‒0.29
*nad6*	59.4	60.6	‒0.52	‒0.52	0.38	0.42
*cytb*	62.9	62.5	‒0.02	‒0.02	‒0.21	‒0.23
*tRNAs*	59.5	60.36	0.02	0.02	0.08	0.07
*rrnA*	59.9	59.8	0.25	0.25	‒0.07	‒0.07

P.M, Peninsular Malaysia.
